# Understory herb layer exerts strong controls on soil microbial communities in subtropical plantations

**DOI:** 10.1038/srep27066

**Published:** 2016-05-31

**Authors:** Kai Yin, Lei Zhang, Dima Chen, Yichen Tian, Feifei Zhang, Meiping Wen, Chao Yuan

**Affiliations:** 1 Institute of Remote Sensing and Digital Earth, Chinese Academy of Sciences, Beijing 100101, China; 2 State Key Laboratory of Vegetation and Environmental Change, Institute of Botany, Chinese Academy of Sciences, Beijing 100093, China

## Abstract

The patterns and drivers of soil microbial communities in forest plantations remain inadequate although they have been extensively studied in natural forest and grassland ecosystems. In this study, using data from 12 subtropical plantation sites, we found that the overstory tree biomass and tree cover increased with increasing plantation age. However, there was a decline in the aboveground biomass and species richness of the understory herbs as plantation age increased. Biomass of all microbial community groups (i.e. fungi, bacteria, arbuscular mycorrhizal fungi, and actinomycete) decreased with increasing plantation age; however, the biomass ratio of fungi to bacteria did not change with increasing plantation age. Variation in most microbial community groups was mainly explained by the understory herb (i.e. herb biomass and herb species richness) and overstory trees (i.e. tree biomass and tree cover), while soils (i.e. soil moisture, soil organic carbon, and soil pH) explained a relative low percentage of the variation. Our results demonstrate that the understory herb layer exerts strong controls on soil microbial community in subtropical plantations. These findings suggest that maintenance of plantation health may need to consider the management of understory herb in order to increase the potential of plantation ecosystems as fast-response carbon sinks.

Forest plantations occupy approximately 200 million ha worldwide and support the increasing local and global demand for wood^1^. These forest plantations have also been considered as potential fast-response carbon sinks that may mitigate the increase in atmospheric CO_2_ concentration^1^,^2^. Since the 1980s, large-scale reforestation and afforestation programs have greatly increased the plantation area in China to approximately 62 million ha. This represents about one-third of the global plantation area and is the greatest plantation area in any country^3^. It has long been appreciated that soil microbes are central to the maintenance of soil fertility and the functioning of terrestrial ecosystems. Identifying the factors that control the patterns of soil microbial communities is important for understanding the development and carbon storage capacity of these plantations^4^. Microbial communities can greatly affect soil nitrogen (N) and carbon (C) cycling^5^ and thereby alter the production of terrestrial communities^6^. The spatial patterns of soil microbial communities have been relatively well documented in natural ecosystem, such as forest and grassland^7^,^8^; however, these spatial patterns have received much less attention in plantation ecosystems, despite their vast planting area and potential as carbon sinks.

With increasing plantation age, different plantation biomass or colonizing understory herb species are frequently associated with different microbial communities^9^. As the development proceeds, there is a greater amount of plant-derived organic matter in the soil as a result of greater plant biomass input into soils^10^,^11^. Such changes in the components of the soil microbial community during vegetation development are well documented^9^,^12^,^13^. A build-up of nitrogen during the initial stages of development often results in a shift in the microbial community from being nitrogen-limited to carbon-limited^5^. This contributes to the rapid accumulation of organic matter during primary plantation development, which can be especially apparent when understory herb species capable of significant nitrogen fixation are dominant^12^,^14^,^15^. Generally, plantation trees may directly impact root-associated soil organisms through resources produced by the roots (root exudates), and the decomposer food web by determining the quantity and quality of the litter that enters the soil^16^.

Several pathways may primarily determine how plantation development affects soil microbes. In the first pathway, increasing above- and belowground plant biomass directly increases carbon availability for soil organisms through litter and root exudates^5^,^16^. During plantation development, this increase in soil carbon availability can enhance soil microbial biomass and activity^17^,^18^. In the second pathway, plantation trees produce indirect effects by driving changes in understory composition. Understory vegetation changes the carbon allocation and also plays an important role in driving the soil microbial community by affecting decomposition, soil nutrient cycling and soil water conservation^19^. An increase in plantation tree cover could suppress the amount of light available for the understory herb community, which also affects carbon allocation as well as soil microbes^9^,^16^. In the third pathway, overstory and understory plants jointly determine the soil moisture, soil pH, and nutrients, which affect soil microbes. Although changes in several specific trophic levels of belowground food webs have been documented in response to understory removal in forest or plantation ecosystems^9^,^12^, data are lacking concerning the relative contributions of these pathways to changes in soil microbes in plantations.

Here, we examined the patterns and drivers of soil microbial communities in *Acacia crassicarpa* plantations by sampling 36 plots in subtropical China; the plots covered three plantation ages and a wide range of abiotic (soil) conditions. We investigated two questions in our study. First, how do understory herb community and soil properties change along plantation age gradient? Second, how do the relative effects of overstory trees (plantation tree biomass and tree cover), soil properties (soil moisture, pH, and soil organic carbon), and the understory herb community (herb species richness and herb aboveground biomass) on the soil microbial community in subtropical plantations? With respect to the first question, we hypothesized that higher plantation tree biomass would result in a decrease in the understory herb biomass and species richness, soil moisture, and soil pH, but an increase in the soil organic carbon content. With respect to the second question, we hypothesized that higher aboveground plantation tree biomass would be associated with fungal-dominated microbial community and high fungi to bacteria ratios due to nutrient-poor environments resulting from the high aboveground tree biomass and soil carbon content and the low soil pH. We also hypothesized that plantation tree biomass would be more important than the understory herb and soil factors because plantation trees directly regulate the soil microbial community through the relative high quantity of root exudates and plant litter returned to soils.

## Results

### Overstory trees, understory herbs, and soil factors of different plantation ages

**Figure 1:**
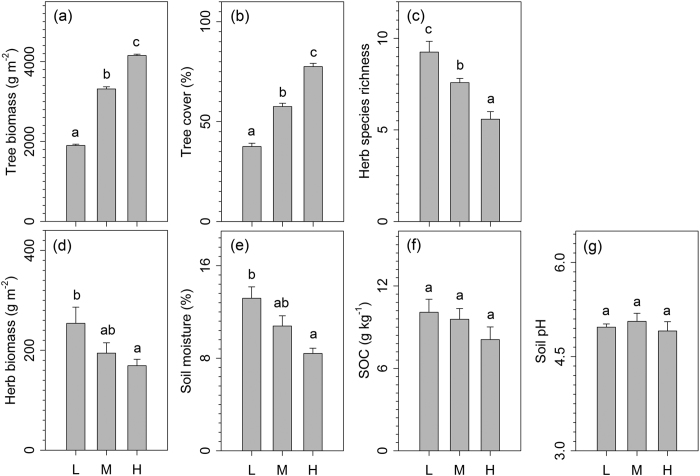
Responses of plantation trees (**a**, biomass; **b**, cover), understory herbs (**c**, species richness; **d**, biomass), and soils (**e**, soil moisture; **f**, soil organic carbon; **g**, soil pH) to plantation age in the subtropical plantations. L, low plantation age; M, medium plantation age; H, high plantation age. Values are means +SE. Within each panel, bars with different letters indicate significant differences among the four community types (one-way ANOVA, *P* < 0.05).

Tree biomass and cover increased with increasing plantation age, i.e. the high plantation age sites had the highest aboveground tree biomass and tree cover (Fig. 1a,b). With respect to the understory plants, the high plantation age had the lowest understory herb biomass and understory herb species richness among the plantation ages (Fig. 1c,d). Similarly, soil moisture decreased with increasing plantation age (Fig. 1e). There was no difference in the soil organic carbon content or the soil pH among the plantation ages (Fig. 1f,g). We also found that the overstory tree plantation biomass and tree cover had negative effects on the aboveground biomass and species richness of the understory herbs (Fig. S1).

### Microbial communities of different plantation ages

**Figure 2:**
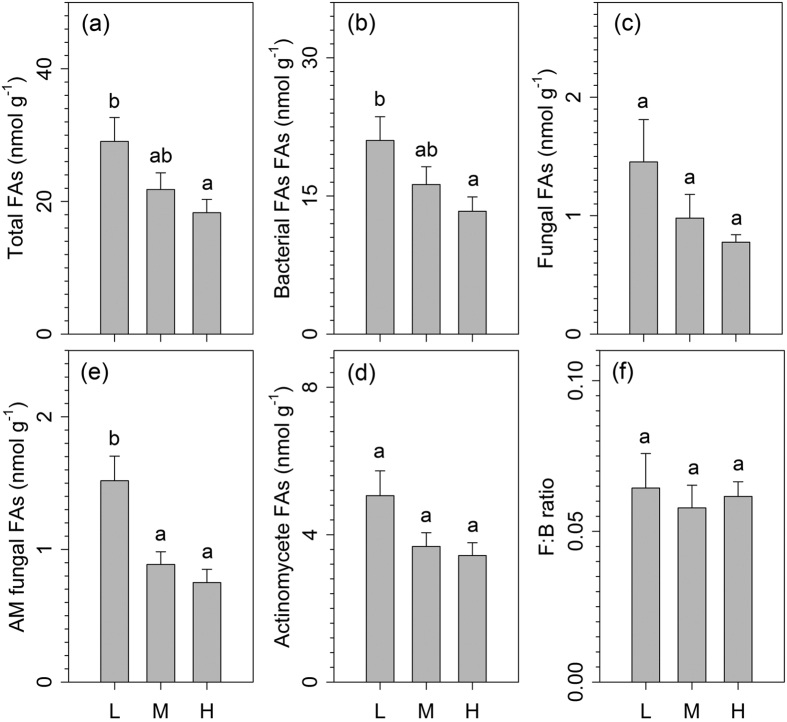
Responses of the soil microbial community to plantation age in the subtropical plantations (**a**–**f**). AM, arbuscular mycorrhizae; B:F ratio, ratio of bacterial FAs to fungal FAs. L, low plantation age; M, medium plantation age; H, high plantation age. Values are means + SE. Within each panel, bars with different letters indicate significant differences among the four community types (one-way ANOVA, *P* < 0.05).

The plantation age changed the total microbial community biomass and community composition (i.e. bacterial biomass, fungal biomass, actinomycete biomass, and AMF biomass) but did not change the community composition of fungi: bacteria ratio. The low plantation age sites were associated with the highest total microbial biomass (29.0 nmol g^−1^ dry soil) while the high plantation age sites were associated with the lowest total microbial biomass (18.3 nmol g^−1^ dry soil) (Fig. 2a). Similarly, the fungal biomass, bacterial biomass, actinomycete biomass, and AMF biomass decreased with increasing plantation age (Fig. 2b–e), whereas the plantation age did not change the F:B ratio (Fig. 2f).

### Relative role of overstory trees, understory herbs, and soil variables in soil microbial community

**Figure 3:**
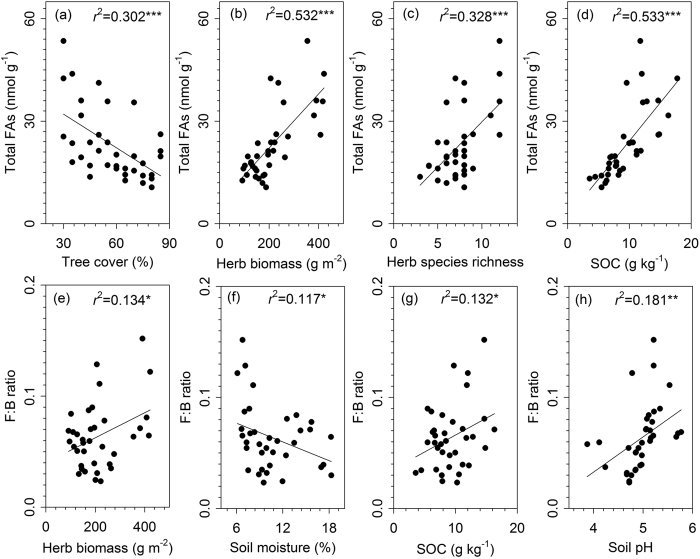
Relationships between the overstory plantation trees, understory herbs and soils, and soil microbial communities across the subtropical plantations. Abbreviations are explained in Table 1. Statistics (*r*^2^ and *P* values) for regression are indicated (*P < 0.05; **P < 0.01; ***P < 0.001).

According to the variation partitioning analyses, the variation in most of the microbial community groups was mainly explained by the understory herb layer (i.e. understory herb biomass and herb species richness) and overstory trees (i.e. aboveground tree biomass and tree cover), while soils (i.e. soil moisture, soil organic carbon, and soil pH) explained a relative small proportion of the variation (Table 1). The variation in the F:B ratio was mainly explained by the soil factors (i.e. soil organic carbon and pH), while the overstory and understory variables explained only a small percentage of the variation (Table 1). The dominant effects of the understory herb layer on the microbial community groups were supported by strong positive relationships between the understory herb biomass and herb species richness, and the microbial groups (Figs 3a–h and S2). We also found that the soil organic carbon content and pH had positive relationships with the microbial community composition (Figs 3a–h and S2).

## Discussion

Our findings showed that the understory herb biomass and herb species richness decreased with increasing plantation age. The results are consistent with our first hypothesis that the understory herb layer will decrease with increasing plantation age. It is generally assumed that light is the limiting resource for the abundance and diversity of understory plants^20^,^21^. In forest ecosystems, the decline in the light supply for the understory is likely to contribute to the declines in understory herb biomass and herb species richness^22^,^23^. In line with these interpretations, the low light availability (indicated by tree cover) for the understory plants in our plantations appears to be the dominant factor limiting the biomass and richness of the herb community with increasing plantation age. The effect of limited light on understory herb biomass and species richness was also observed in forest and grassland ecosystems. For instance, using experimental grassland plant communities, Hautier *et al.*^24^ found that the addition of light to the grassland understory prevented the loss of biodiversity caused by eutrophication. In addition, the strong ability of trees to acquire other resources (e.g. water) is another principal constraint on understory herbs during plantation development^20^,^25^. Overall, the understory light, soil water availability, and soil nutrients jointly determine the decline in the herb biomass and species richness during early plantation development.

In general, plantation soils tend to sequester more carbon during early plantation development due to enhanced biomass productivity, litter fall, formation of stable aggregates, and the residence time of carbon^26^. Plantation systems have considerable plant biomass allocation to belowground soil carbon and help mitigate the greenhouse effect by reducing carbon emissions^27^,^28^. Our finding that soil organic carbon did not increase with plantation age did not support our first hypothesis that plantation soils tend to sequester more carbon with increasing age. This result is inconsistent with previous studies, which showed that soil organic carbon increased with plantation age^29^,^30^. For example, in subsurface soils of *H. alchorneoides* and *V. guateamalensis*, the corresponding soil organic carbon increase was 33, 36 and 64% in plantations aged 1, 3 and 6 years, respectively^31^. Our results are consistent with some reports, which showed that plantation age had no effect on soil organic carbon^32^,^33^. This lack of consistency with previous reports might be explained by that: 1) an increase in respiration (including both heterotrophic and autotrophic respiration) during early plantation development, and the plant material was only slowly incorporated into the soil in this subtropical plantation; 2) more carbon will be lost from soil than carbon input from plants at the initial growth stage. The observed soil organic carbon accumulation in previous study probably due to young stand age, disturbance from site preparation, and soil spatial heterogeneity.

Our observations in the subtropical plantations provide evidence that plantation age can reduce soil microbial groups (e.g., bacterial biomass, fungal biomass, AMF biomass, and actinobacterial biomass). These results are inconsistent with our second hypothesis that the biomass of soil microbial groups will increase with plantation age. The decline in the biomass of the microbial groups with increasing plantation age is inconsistent with previous reports, which showed that increases in plantation age or tree productivity led to an increase in the composition of the belowground communities^34^,^35^. Although several other studies indicated that plantation age or tree productivity did not affect soil microbial communities^36^,^37^, our study supported other forest studies, which found that increases in plantation productivity negatively affected soil microbial functional groups^12^,^38^. In *Eucalyptus urophylla* plantations, for example, an increase in plantation age resulted in reduced microbial biomass^12^. The negative effects of increasing plantation age on soil microbial communities in this subtropical plantation indicate that the availability of nutrients in plantations, as mediated by soil microbes, could also be reduced^5^, potentially resulting in a long-term reduction in ecosystem productivity.

We found that the fungal to bacterial biomass ratio of the microbial community did not change with increasing plantation age. This result did not support our second hypotheses that plantation age would shift the microbial community from bacteria-dominated to fungi-dominated^12^. These findings are consistent with those from other forest ecosystems, which indicated that the ratio of fungal to bacterial biomass did not change with increasing plantation age^13^. These responses might be explained by the fact that there were no changes in the quantity of plant materials available to the belowground organisms. This stability in the bacterial- and fungal-based soil food webs with respect to plantation age may result in subtropical plantations being stable in response to changes in land use or climate.

In our study, the variation in the biomass of the soil microbial groups in the subtropical plantations was mainly explained by the overstory trees and the understory herbs rather than by the soil factors; the overstory trees affected the soil microbes mainly through the understory and soil factors. These results are partly consistent with our third hypothesis that overstory tree biomass would be more important than the understory herb and soil factors. The composition of plant litter and root exudates differs among plantation age, and changes in plantation biomass are likely to alter the quality of the resources supporting the belowground communities^39^. We suspect that the microbial communities in our research benefitted more from the understory herbs than from the plantation trees because: 1) the understory herbs provided a higher quantity of resources to the soil microbial communities^12^,^20^; 2) the higher N-exploitative plant species of the understory herbs produced litter with a lower C:N ratio^16^, which may have enhanced the biomass of the soil microbial community groups^9^; and 3) the understory herbs may have provided direct physical protection for the soil microbes by producing more plant litter^20^. In the present study, the biomasses of the soil microbial community components were positively related to the understory herb biomass and species richness, which is consistent with previous findings, suggesting that plantation trees affect belowground communities mainly through understory productivity and soil nutrients^40^. Most belowground organisms are heterotrophs that rely on the plant-derived residues that enter the soil via leaf and root litter and rhizodeposition^39^,^40^. Thus, the decline in the biomass or abundance of microbes in response to plantation age likely resulted from decreases in the quantity and quality of understory herb materials entering the soil, rather than plantation tree materials.

In addition to the understory herb properties (i.e., biomass and species richness), the relatively low percentage of the variation in the soil microbial communities explained by the soil factors in the subtropical plantations may be due to the relatively minor effects of plantation age on soil moisture, soil organic carbon and pH. Many previous studies found that changes in soil properties partially explained the subsequent changes in the soil microbial community composition^8^,^41^. In this subtropical plantation, we found that the soil organic carbon and soil pH also accounted for a significant percentage of the variation in the microbial communities. These results are consistent with previous reports, which indicated that total biomass, bacterial biomass, and fungal biomass were affected by soil organic carbon and soil pH at the regional scale and ecosystem level^41^,^42^. Overall, our findings indicate that plantation age and understory plant may greatly affect soil microbial communities and may even lead to a loss of soil nutrients from a plantation ecosystem.

## Materials and Methods

### Study area and experimental design

The field studies did not involve endangered or protected species, and no specific permissions were required for these locations. This study was conducted in *Acacia crassicarpa* plantations in Xiamen city, a critical part of the plantation region in China. The study area is located at 24.42°–24.91°N latitude and 117.83°–118.42°E longitude. The site is characterized by a typical southern climate of subtropical monsoons and a laterite soil. In this region, there is a distinct wet and dry season. The wet season starts in March and ends in September, and the dry season starts in October and ends in February. The mean annual precipitation (MAP) is about 1143.5 mm and the mean annual temperature (MAT) is about 20.7 °C, with the lowest mean monthly temperature in January and the highest in July. We established 12 sites in July 2009 across a biomass gradient in Southeast China. These sites cover three classes of plantation ages: 3–4 years (low plantation age): 6–7 years (medium plantation age), and 10–11 years (high plantation age). The dominant understory vegetation species include *Dicranopteris dichotoma*, *Adiantum capillus-veneris*, *Lonicera japonica*, *Woodwardia japonica*, and *Miscanthus floridulus*.

### Field sampling and measurements

At each site, aboveground tree biomass and understory herbaceous biomass were determined. The aboveground tree biomass in each site was estimated in a 20 × 20-m topographically uniform area. The diameter at breast height (DBH) and height (H) of the representative trees were recorded. Individual aboveground tree biomass (TB_*ind*_) was calculated using the following equation: TB_*ind*_ = 4.8301* ((DBH)^2^H)^0.4394 ^1. The aboveground tree biomass value for each site was obtained by multiplying the individual tree count and aboveground tree biomass. We also determined the tree cover (%) of each site using a spherical densiometer (Forest Densiometers, Bartlesville, OK). The understory herb biomass was determined in three 1 × 1-m quadrats located randomly within the 20 × 20-m area. For each quadrat, the aboveground biomass of the live understory herb was clipped at ground level. All herbaceous materials in each quadrat were oven-dried at 65 °C for 48 h and weighed. The total dry mass, number of species, and biomass of each species were used to estimate the understory herbaceous biomass and species richness of each quadrat.

Soil samples were obtained by randomly collecting five 3-cm-diameter soil cores from a depth of 0 to 20 cm in each of the three 1 × 1-m quadrats. The three cores from each quadrat were mixed *in situ* to form one composite sample. After gentle homogenization and removal of roots, the moist soil was sieved through a 2-mm mesh and separated into two parts. One part was maintained fresh for measuring soil moisture. The other was air-dried to determine the soil pH and soil organic carbon content. A subsample of 20 g of moist soil was oven-dried at 105 °C for 24 h to determine the soil moisture. Soil pH was measured in a 1:2.5 (soil:water) suspension. The soil organic carbon content was determined using the Walkley–Black modified acid-dichromate FeSO_4_ titration method. All results are expressed on a dry weight basis.

### Soil microbial community

The soil microbial community was assessed by analysis of phospholipid fatty acids (PLFAs)^43^. The resultant fatty acid methyl esters were separated, quantified, and identified using capillary gas chromatography (GC). Qualitative and quantitative PLFA analyses were performed using an Agilent 6890 gas chromatograph (Agilent Technologies, Palo Alto, CA, USA) and the MIDI Sherlock Microbial Identification System (MIDI Inc., Newark, DE, USA). The abundance of each individual PLFA in a given sample was expressed as PLFA nmol g^−1^ dry soil against an internal standard (methyl ester C19:0, Matreya Inc., State College, PA, USA). PLFAs specific to bacteria (i14:0, a15:0, i15:0, i16:0, a17:0, i17:0, 16:1ω7c, 17:1ω8, 18:1ω9, 18:1ω7c, cy17:0, and cy19:0), fungi (18:2ω6, 9), arbuscular mycorrhizal fungi (16:1ω5c), and actinobacteria (10Me 16:0, 10Me 17:0, and 10Me 18:0) were used to determine the concentrations of these microbial groups and to calculate the ratio of bacterial biomass to fungal biomass (F: B ratio)^44^,^45^. All results are expressed on a dry weight basis.

### Statistical analyses

All statistical analyses were performed using R version 2.15.1 (R Development Core Team 2009). First, soil microbial components and structure variables, overstory tree variables (aboveground tree biomass and tree cover), understory herbaceous variables (herb biomass and herb species richness), and soil properties (soil moisture, soil pH, and soil organic carbon) were compared between the three classes of plantation age using one-way ANOVA. The data were transformed (natural logarithms) before analysis to improve normality. Second, we used variation partitioning analyses to determine the relative effects of the overstory trees, understory herbaceous layer, and soil variables on the soil microbial variables using the calc.relimp function in the “relaimpo” package^46^. Finally, the relationships between the three potential explanatory variables and soil microbial variables were further examined using linear or quantic regression. The Akaike information criterion (AIC) was used to select the most appropriate model.

## Additional Information

**How to cite this article**: Yin, K. *et al.* Understory herb layer exerts strong controls on soil microbial communities in subtropical plantations. *Sci. Rep.* **6**, 27066; doi: 10.1038/srep27066 (2016).

## Supplementary Material

Supplementary Information

## Figures and Tables

**Table 1:** The percentage variation (as determined by linear models) in the soil microbial communities explained by the trees, understory plant communities, and soils of the subtropical plantations.

Microbes	Overstory trees		Understory herb		Soils			Residuals (%)
	TB (%)	TC (%)	HSR (%)	HB (%)	SM (%)	SOC (%)	pH (%)	
Total FAs (nmol g^−1^)	6.3	14.3	15.0	20.1	0.7	11.7	0.4	31.6
Bacterial Fas (nmol g^−1^)	5.8	10.7	13.9	20.7	1.0	12.3	0.5	35.1
Fungal FAs (nmol g^−1^)	4.2	10.5	9.8	19.4	2.4	9.3	7.2	37.3
AM fungal FAs (nmol g^−1^)	8.7	19.6	12.9	11.3	1.6	7.3	0.3	38.3
Actinomycete FAs (nmol g^−1^)	6.7	15.8	13.4	17.0	0.4	8.0	0.8	37.9
B:F ratio	1.8	2.2	1.4	12.2	7.1	13.3	27.6	34.4

AM, arbuscular mycorrhizae; B:F ratio, ratio of bacterial FAs to fungal FAs; TB, aboveground biomass of overstory trees; TC, overstory tree cover; HSR, understory herb species richness; HB, aboveground biomass of understory herbs; SM, soil moisture; SOC, soil organic carbon.
