# Supplementary material for: Understory herb layer exerts strong controls on soil microbial communities in subtropical plantations
Source: Sci Rep. 2016 May 31;6:27066. doi: 10.1038/srep27066 (PMC4886681; doi:10.1038/srep27066)
Supplement: Supplementary Information [file srep27066-s1.doc]

Title: Understory herb layer exerts strong controls on soil microbial communities in subtropical plantations

Kai Yin^1†^, Lei Zhang ^1†^, Dima Chen^2*^, Yichen Tian^1*^, Feifei Zhang^1^, Meiping Wen^1^, Chao Yuan^1^

^1^Institute of Remote Sensing and Digital Earth, Chinese Academy of Sciences, Beijing 100101, China

^2^State Key Laboratory of Vegetation and Environmental Change, Institute of Botany, Chinese Academy of Sciences, Beijing 100093, China;

^†^These authors contributed equally to this work.

^*^Corresponding author:

Dima Chen, E-mail: [chendima@ibcas.ac.cn](mailto:yfbai@ibcas.ac.cn); or Yichen Tian, E-mail: tianyc@radi.ac.cn

**Supplementary Information**

**Results**

**Figure S1** Relationships between overstory plantation trees and understory herbs across the subtropical plantations. Statistics (*r*^2^ and *P* values) for regression are indicated (**P* < 0.05; ***P* < 0.01; ****P* < 0.001).

**Figure S2** Relationships between tree cover, herb biomass, herb species richness and SOC (soil organic carbon) content, and the soil microbial communities across the subtropical plantations. Abbreviations are explained in Table 1. Statistics (*r*^2^ and *P* values) for regression are indicated (**P* < 0.05; ***P* < 0.01; ****P* < 0.001).
